# How will air quality effects on human health, crops and ecosystems change in the future?

**DOI:** 10.1098/rsta.2019.0330

**Published:** 2020-09-28

**Authors:** Erika von Schneidemesser, Charles Driscoll, Harald E. Rieder, Luke D. Schiferl

**Affiliations:** 1 Institute for Advanced Sustainability Studies, Berlinerstrasse 130, 14467 Potsdam, Germany; 2 Department of Civil and Environmental Engineering, Syracuse University, Syracuse, NY 13244, USA; 3 Institute of Meteorology and Climatology, University of Natural Resources and Life Sciences, Vienna, Gregor-Mendel Strasse 33, 1180 Vienna, Austria; 4 Lamont-Doherty Earth Observatory, Columbia University, Palisades, NY 10964, USA

**Keywords:** air pollution, future climate projections, ozone, particulate matter, health and ecosystem effects, food security

## Abstract

Future air quality will be driven by changes in air pollutant emissions, but also changes in climate. Here, we review the recent literature on future air quality scenarios and projected changes in effects on human health, crops and ecosystems. While there is overlap in the scenarios and models used for future projections of air quality and climate effects on human health and crops, similar efforts have not been widely conducted for ecosystems. Few studies have conducted joint assessments across more than one sector. Improvements in future air quality effects on human health are seen in emission reduction scenarios that are more ambitious than current legislation. Larger impacts result from changing particulate matter (PM) abundances than ozone burdens. Future global health burdens are dominated by changes in the Asian region. Expected future reductions in ozone outside of Asia will allow for increased crop production. Reductions in PM, although associated with much higher uncertainty, could offset some of this benefit. The responses of ecosystems to air pollution and climate change are long-term, complex, and interactive, and vary widely across biomes and over space and time. Air quality and climate policy should be linked or at least considered holistically, and managed as a multi-media problem.

This article is part of a discussion meeting issue ‘Air quality, past present and future’.

## 1. Introduction

Air quality and climate change are inextricably linked. Not only do some air pollutants have a direct effect on radiative forcing (e.g. ozone (O_3_) and particulate matter (PM), including black carbon) and thereby climate change, but changing climate can affect air quality. Furthermore, given the commonality in emissions sources, mitigation options will likely affect both air quality and climate change. For example, fossil fuel burning—whether for energy, transport, or otherwise—emits both the greenhouse gas carbon dioxide (CO_2_), as well as air pollutants such as nitrogen oxides, sulfur dioxide, mercury and particulate matter. Agriculture is a significant source of the greenhouse gas methane, as well as air pollutant emissions of particulate matter and reduced nitrogen. Emissions of reduced nitrogen, which include ammonia, ammonium and organic nitrogen, are increasing and are projected to continue to increase in importance globally, due to decreases in emissions of nitrogen oxides coupled with increases in agricultural and other sources of reduced nitrogen [1]. Reduced nitrogen can be an important component of PM, but also comprises an increasingly larger fraction of atmospheric nitrogen deposition, with implications for croplands and ecosystems. Mercury, an important hazardous air pollutant, has multiple emission sources in addition to fossil fuels (mostly coal). The most important of these are artisanal and small scale gold mining, cement production and non-ferrous metal [2]. Atmospheric processes also link air quality and climate. For example, concentrations of methane affect the concentrations of ozone in the troposphere, which serves as not only an air pollutant, but also a greenhouse gas. Furthermore, the lifetime of the greenhouse gases and air pollutants in the atmosphere determines the temporal and spatial scale of their effects. Changes in the composition of the atmosphere are generally the result of long-term processes such as the slow evolution in emissions associated with increases in population; increases in fuel, food and material consumption; changes in technology or the gradual implementation of air pollution control policies. However, extreme events can cause abrupt changes in air quality, such as volcanic eruptions or the recent (temporary) decrease in emissions associated with the global COVID-19 pandemic. These linkages demonstrate some potential co-benefits and trade-offs that should be considered when formulating air quality and climate change policies [3–5].

A detailed overview of chemistry–climate interactions is provided in Archibald *et al*. [6], or previous review articles [7–10]. In this paper, we synthesize the current understanding of the influence of climate change on future air pollution effects. Of particular relevance is the concept of a ‘climate penalty’, whereby future climatic conditions in a warming world will exacerbate the challenge in reaching air quality targets or standards. Greater emissions reductions will be necessary to attain the same air quality target in a warmer climate in comparison to a stationary climate scenario [11,12]. The climate penalty has been widely established in the literature for ozone, due to the role of photochemistry (sunlight) in ozone production, whereby increases in air temperatures enhance ozone formation [13–16]. Climate change effects are less understood for PM, due to the diversity of particulate matter components, formation and removal mechanisms, and the role of phenomena such as wildfires which are intensified under climate change [17–21]. Changing climate not only influences atmospheric processes, but also emissions. For example, higher air temperatures will increase evaporative emissions of anthropogenic volatile organic compounds, as well as emissions of biogenic volatile organic compounds [22,23] and ammonia. Furthermore, emissions of methane from wetland ecosystems, permafrost environments and other critical ecosystems are likely highly sensitive to climate change [24]. Finally, policies implemented to mitigate greenhouse gas emissions have considerable potential to improve future air quality. There is considerable potential for co-benefits whereby either climate or air quality policies could greatly improve environmental quality if interactive effects are considered [3–5]. The air quality co-benefits from climate policies may in some cases even exceed the direct climate benefits (e.g. [25]). Understanding these feedbacks and interactions sufficiently to accurately depict them in models and thereby improve future projections is a critical need to inform and guide coupled management of air quality and climate.

Future projections are most often investigated through the use of scenarios. Alternatively, case studies of extreme events that might depict future conditions are also used for understanding and quantifying possible future effects (e.g. [26–29]). In the current context of the Anthropocene, human activity has a dominating influence on climate and the environment [30]. Here, we review the state of science that aims to quantify how expected changes in future air quality affect human health, ecosystems and food security. Climate change is an important driver of future air quality. However, additional, often less considered factors include changes in population demographics, adaptation measures and feedbacks across the sectors of human health, agricultural systems and ecosystems.

## 2. Scenarios used in future projections

A variety of climate scenarios are commonly used, especially in the context of changes in air quality and effects on human health and crop yields. Among the most common are the representative concentration pathways (RCPs) [31]. These four scenarios were designed for the climate modelling community to span the range of plausible emissions futures and the associated changes in radiative forcing in support of the Fifth Intergovernmental Panel on Climate Change (IPCC) Assessment Report [32]. The scenarios include land use changes and emissions of air pollutants and greenhouse gases extending to 2100, with the endpoints of the four scenarios associated with the amount of radiative forcing. The scenarios range from RCP2.6 (2.6 W m^−2^) which represents a scenario that would limit global temperature increase consistent with a 2°C target, and requires greater nuclear and renewable energy, as well as carbon capture and storage, to RCP8.5 (8.5 W m^−2^) which represents a business as usual scenario with continued reliance on fossil fuels and no specific policies to limit greenhouse gas emissions, with intermediate scenarios for RCP4.5 (4.5 W m^−2^) and RCP6.0 (6.0 W m^−2^). While the scenarios include air pollutant emissions, they do not represent the possible range of air pollution pathways, instead generally assuming stricter emission controls on air pollutants over time in all four scenarios [33]. The main difference among the RCPs is the pathway for methane emissions which increases under RCP8.5, due to the projected increase in livestock and rice production, and will, therefore, have the largest impacts on the changes in the temporal and spatial pattern of concentrations of tropospheric ozone [34]. Nevertheless, these scenarios are used in many of the studies evaluating developments in future air quality effects on human health.

Additional sets of scenarios used by some studies included in this review are the Special Report on Emissions Scenarios (SRES) published in 2000 by the IPCC for future assessments of climate change and possible response strategies [35], or the shared socioeconomic pathways (SSPs) that provide a set of alternative reference assumptions about future socioeconomic development in the absence of climate policies or climate change, that complement the radiative forcing pathways provided by the RCPs [36]. A number of studies evaluate emissions scenarios for current legislation (CLE) and maximum technically feasible reduction (MFR) as provided in the ECLIPSE v5a (Evaluating the Climate and Air Quality Impacts of Short-Lived Pollutants) emissions inventory [37]. For both the SSPs and the scenarios from ECLIPSE, this information has only become available recently, and therefore the literature published using these scenarios is limited. Finally, a handful of studies also evaluate policies or policy proposals that are linked to local or regional legislation.

## 3. Future air quality impacts

**Figure 1.**
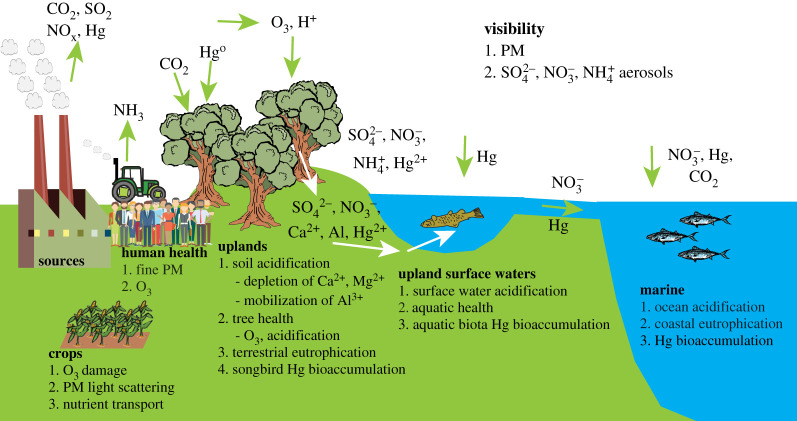
Conceptual diagram showing sources of air pollution and potential effects on human health, agroecosystem function and ecosystem structure and function. Human health is impacted by fine particulate matter and ozone. Crops are largely affected by ozone, but also light scattering from particulate matter and nutrient transport. Ecosystem effects include uplands, freshwaters and coastal and marine waters and involve ozone impacts, acidification, eutrophication and mercury effects. Note it also envisions linkages among human health, agriculture and ecosystem sectors. (Online version in colour.)

Although future air quality will likely have adverse effects on human health, crops and ecosystems that will be exacerbated under climate change, few studies have examined the linkages of impacts across multiple sectors (figure 1). Studies tend to evaluate these broad types of effects in isolation. There is substantial literature on projected estimates of the effect of future air quality on human health, from both global and regional perspectives. The papers included in this review for the human health effects reflect the past 6 years, extending back through 2014. To this end, we performed an indexed search in the Web of Science (index terms: air pollution and health, air quality and health, ozone and health, PM and health). Further, we included studies not identified directly by the Web of Science search but cited in studies emerging in the identified literature. For an overview of earlier multi-model studies and reviews, the reader might see [38–41]. Given the impact focus, the studies included here explicitly focus on changes in future health burdens (such as changes in mortality, disease cases or monetized effects). The rich body of literature focusing on future air quality but not addressing changing health burdens has been omitted. Similarly, those studies addressing changes in health burdens linked only to changes in climate or other factors in which air quality changes are not quantified are also not included. There is far less literature on projections on effects of future air quality on crop yields, thus studies as far back as 2009 are included. All studies available through reasonable search which quantifiably related air quality to future crop production were included. The existing studies tend to focus on four main crops: wheat, maize, rice and soya beans. Both sets of literature that evaluate the effects of future air quality on human health and crops tend to use scenarios as outlined in the previous section, with the majority of studies based on the RCPs. By contrast, studies that project changes in the structure and function of ecosystems under future air quality and climate generally have not employed these types of scenarios (but see [42–46]), and those that have generally focus on climate impacts rather than air quality. There are a number of reasons for this. First, there are a broad range of ecosystem effects of air pollution, such as soil and freshwater acidification, mercury contamination, eutrophication of terrestrial and marine ecosystems, visibility, among others, each requiring separate modelling efforts. Ecosystem impacts often require simulation of linked hydrologic, biogeochemical and biotic processes involving the dynamics of multiple elements within a watershed or water body to project responses (e.g. [47,48]). Second, ecosystem disturbance by air pollution generally impacts large spatial regions. Generally, dynamic models of ecosystem effects of air pollution have not been well validated across the heterogeneous landscapes, which encompass a range of topographic, meteorological, hydrologic, edaphic and vegetation conditions. Finally, the developed future climate scenarios do not include some of the key air pollutants critical to ecosystems, such as mercury. Given this, we provide observations on the scope of air pollution effects on ecosystems and how these responses are altered by climate, as well as three case studies that demonstrate the scope of these effects.

### (a) Human health

An overview of the 47 studies that examine future projections of changes in air quality related health burdens and mortality, as well as other human health indicators, between 2014 and early 2020 is given in table 1. Here, we provide a brief overview and some highlights from these studies, starting with global assessments. A couple of global studies focused solely on aerosol related health burdens [51,56], while most evaluated health effects of changes in both ozone and PM (generally PM2.5 rather than PM10, particles with an aerodynamic diameter of 2.5 µm or less and 10 µm or less, respectively, owing to the greater relevance for adverse health effects). The majority of global scale studies also provide detailed regional information for the USA, Europe and/or Asia. The overview of literature with a global perspective is then complemented by brief insights into regionally focused projections, including examples highlighted from each region. Global projections that include regional detail are not included in the regional sections. No regional studies were identified that examined scenarios of air quality and the associated health effects in Central and South America or Africa. In a nutshell, studies agreed that emission trajectories attaining 2°C climate targets, clean energy/transportation scenarios, and low to moderate emission scenarios among the RCPs/SSPs will lead to improved ambient air quality and reduced health burdens, while air quality will further deteriorate and increase health burdens under business as usual trajectories and high emission RCPs/SSPs. Studies also found that the monetized health gains of reduced PM and ozone burdens exceeded the implementation costs of climate protection and emission reduction measures.

**Table 1.** Summary of studies published since 2014 on future changes in air quality effects on human health. Only papers that specifically quantify health effects from air pollution are included. Health unit abbreviations are as follows: ED, excess deaths; AD, avoided deaths; %, per cent change in mortality; $, economic cost; O, other (such as hospital admissions, years of life lost, etc.).

publication	region	scenario(s)	base year(s)	future year(s)	ozone	PM2.5^a^	health units
Global							
Likhvar *et al*. [49]	Global, Europe, Ile-de-France region	CLE, MFR	2010	2030, 2050	X	X	ED,%, O
Lelieveld *et al*. [50]	Global, WHO Regions	BAU, CLE	2010	2025, 2050	X	X	ED,%
Morita *et al*. [51]	Global	RCP4.5 aviation sector; ref scen. 4.8× increase fuel burn; fuel efficiency goal of 2% per annum by 2050 and 2.7× increase fuel burn; alt 2nd scen. sulfur-free fuel and max 8% aromatic content	2006	2050		X	ED, AD
Anenberg *et al*. [52]	11 major vehicle markets	NO_x_ emission inventories and emission factors, vehicle activity projections through 2040, Emission limits 2015 and 2040, Euro 6/VI 2040, Next Generation (NextGen) 2040	2015	2040	X	X	ED
Shindell *et al*. [53]	Global	US emission reductions (RCP8.5 baseline), clean transport, clean energy scenarios; consistent with 2° target		2030	X	X	AD,$
Silva *et al*. [34]	Global	All RCPs	2000	2030, 2050, 2100	X	X	AD,ED
Silva *et al*. [54]	Global	RCP8.5, climate change effect isolated (versus emissions)	2000	2030, 2100	X	X	ED,%
Markandya *et al*. [55]	Global and selected world regions	4 temp scenarios (no climate policy, domestic/natl level targets, 2° target, 1.5° target), also follows SSPs	2005	2020–2050	X	X	ED,%, $
Partanen *et al*. [56]	Global	RCP2.6, RCP4.5, RCP8.5, 2 alternative aerosol emission scenarios	2005	2030		X	ED
Shindell *et al*. [57]	Global	2° scenario, 2° scenarios with no negative emissions; linked to RCP2.6		2020–2100	X	X	ED,AD,$
Vandyck *et al*. [58]	Global and selected world regions	only current climate change policies, NCDs, 2° target, BAT, SLE, FLE	2010	2030, 2050	X	X	AD, $
Asia and Australia							
Physick *et al*. [59]	Sydney	A2	1996–2005	2051–2060	X		%, ED
Goto *et al*. [60]	Japan	RCP4.5	2000	2030		X	ED
Lee *et al*. [61]	South Korea (7 cities)	All RCPs	1996–2005, 2001–2010	2016–2025, 2046–2055	X		%
Qin *et al*. [62]	China	synthetic natural gas development strategy, using ECLIPSE_V5a_CLE	2013	2020		X	ED
Yang *et al*. [63]	China	PV capacity China Renewable Energy Roadmap 2050, using ECLIPSE_V5a_CLE	2000	2030		X	ED
Xie *et al*. [64]	Asia	SSP2, SSP3,	2005	2050	X	X	ED, $
Chen *et al*. [65]	China (104 cities)	RCP4.5, RCP8.5, population change SSPs	2013–2015	2053–2055	X		%, ED
Westervelt *et al*. [66]	China	CLE/MFR +RCP8.5	2015	2050	X		ED
Permadi *et al*. [67]	Southeast Asia	BAU, reduced PM emissions for Indonesia and Thailand, others RCP8.5	2007	2030		X	ED
Hong *et al*. [68]	China	RCP4.5	2006–2010	2046–2050	X	X	ED
Li *et al*. [69]	China, South Korea, Japan, USA (only China climate policy)	no policy versus 3%, 4%, 5% CO_2_ intensity reductions per year	2015	2030	X	X	AD
United States of America							
Chang *et al*. [70]	Atlanta metropolitan area (20 counties)	A2	1999–2004	2041–2070	X		O
Kim *et al*. [71]	USA	RCP4.5, RCP8.5	2001–2004	2057–2059	X		ED
Thompson *et al*. [72]	USA	US Regional Energy Policy climate policies (clean energy standard, transportation, cap and trade)	2006	2030	X	X	$
Driscoll *et al*. [73]	USA	2 bipartisan policy center and 1 natural resources defense council scenario, linked to FF power plants	2013	2020	X	X	AD, O
		Annual Energy Outlook for 2013 as reference scenario					
Fann *et al*. [74]	USA	RCP 8.5, RCP6.0	1995–2005	2025–2035	X		ED, O, $
Sun *et al*. [75]	USA	RCP8.5	2002–2004	2057–2059	X	X	ED
Thompson *et al*. [76]	Northeast USA	economy-wide cap and trade, clean energy standard (electricity sector only)	2006	2030	X	X	$
Buonocore *et al*. [25]	USA	policy resembling the US Environmental Protection Agency's Clean Power Plan		2020		X	ED, $, O
Wilson *et al*. [77]	USA (94 urban areas)	RCP6.0—only biogenic emissions change	1995–2005	2025–2035	X		ED, %
Alexeeff *et al*. [78]	USA	RCP8.5	2000	2050	X (summertime)		%
Zhang *et al*. [79]	USA	emissions RCP4.5, sectoral RCP4.5 (industry, residential or energy), Climate RCP4.5/RCP8.5		2050	X	X	AD, %, $
Stowell *et al*. [80]	USA	RCP4.5, RCP8.5	2001–2004	2055–2059	X		ED
Buonocore *et al*. [81]	Massachusetts	carbon fee-and-rebate bill	2017	2040	X	X	AD, $
Abel *et al*. [82]	Eastern USA	A2 + heat-driven adaptation (building energy demand and power sector)	July 2011	July 2069	X	X	ED, $
Achakulwisut *et al*. [83]	Southwest USA	RCP2.6, RCP8.5 (drought conditions)	1996–2015	2076–2095		X	ED, %, O
Ou *et al*. [84]	USA	on-the-books air pollutant emissions and energy regulations, 50% and 80% CO_2_ emission reduction by 2050, faster technology cost reductions for nuclear and CCS technologies	2010	2050		X	$
Saari *et al*. [85]	USA	BAU; POL4.5 POL3.7	BAU	2050, 2100	X	X	ED, %, O
Achakulwisut *et al*. [20]	Southwest USA	RCP4.5, RCP8.5, increasing aridity	1988–2005	2050, 2090		X & coarse dust (PM2.5-PM10)	ED, %, $, O
Wolfe *et al*. [86]	USA	mobile source reductions	2011	2025		X	$
Martinich & Crimmins [87]	Sectors of the USA	RCP4.5, RCP8.5		2050, 2090	X		ED, $, O
Zhao *et al*. [88]	California	deep decarbonization (DD1 and DD2)	2010	2050	X	X	AD, $
Garcia-Menendez *et al*. [17]	USA	POL4.5, POL3.7, no policy reference scenario	BAU, 2000	2050, 2100	X	X	AD, O
Europe							
Schucht *et al*. [89]	Europe	2 Global Energy Assessment scenarios: no climate policy; climate mitigation limiting global temperature increase to 2°C by 2100	2005	2050	X	X	ED, %, $, O
Geels *et al*. [90]	Europe	RCP4.5	2000–2009	2050–2059, 2080–2089	X	X	ED
Tarín-Carrasco *et al*. [91]	central and southern Europe	RCP8.5	1996–2015	2071–2100	X	X (PM10)	ED, %, $, O

^a^Size fraction is PM2.5 unless otherwise noted.

A broad range of health metrics and health endpoints are used in air quality impact studies. Among the most prominent are *excess deaths*, defined as the increase in the mortality rate (number of deaths) due to the environmental factor of interest, or conversely *avoided deaths*, defined as the mortality rate (number of premature or ‘untimely’ deaths of people younger than a defined age threshold) avoided by interventions (e.g. [92]). Besides those metrics, modified mortality measures taking an individual's remaining life expectancy into account are frequently used. These include e.g. the number of *years of life saved*, defined as the remaining life expectancy at the point of each averted death, and conversely, *years of life lost*, defined as the lifespan lost due to premature mortality attributable to an environmental effect (e.g. [93]). Besides these mortality indices long-term health effects described through *diverse morbidity metrics* are frequently used in impact studies, including cardiovascular, respiratory or neurological endpoints, hospitalization rates, cancer, diabetes and negative pregnancy/birth outcomes (e.g. [94]). Mortality (and morbidity) resulting from ambient air pollution is also frequently monetized to quantify the benefits added by a new policy, with the most frequent economic metric used being the *value of statistical life* (with the valuation extended to incorporate morbidity effects), placing a monetary value on reductions in the risk of a fatality (e.g. [95]). Here, we focus results on mortality in terms of excess or avoided deaths whenever possible as it is one of the most common metrics used across studies.

#### (i) Global projections

To provide a broader context of how air pollution and the global burden of disease might develop in the future, the work of Lelieveld *et al*. [50] provides a good starting point. These authors investigated the contribution of outdoor air pollution sources to premature mortality on a global scale for the recent past and first half of the twenty-first century considering a business as usual scenario, assuming only currently agreed legislation (CLE) would be implemented to affect future air pollution emissions. Their estimate of global premature mortality attributed to air pollution in 2010 was 3.30 million deaths (142 000 from ozone, the remainder from PM2.5), considering a global population of 6.8 billion people. They projected an increase in air pollution attributed premature mortality to 6.6 million deaths in 2050 (358 000 from ozone, all others from PM2.5) [50]. While these premature deaths were dominated by the fraction occurring in Asia, increases were also projected for Europe and the Americas, largely in urban areas. Finally, it was estimated that the per capita mortality rate, which was 50% higher in urban versus rural environments in 2010, is expected to increase to nearly 90% higher in urban versus rural environments by 2050 [50]. One caveat to note with this study (and other global studies) is the mismatch in the spatial resolution of the atmospheric model used to calculate pollutant burdens and the resolution of present/future population datasets used along with these burdens in the exposure response functions determining mortality. This offset can be partially accounted for by aggregating the anthropogenic emission data to the model grid and by updating the exposure response functions for the use of annual mean concentrations to estimate mortalities [96].

By comparison, Likhvar *et al*. [49] also evaluated a scenario of currently agreed legislation, as well as a scenario of maximum feasible reductions (MFR) [97] to quantify premature deaths attributed to changes in PM2.5 and ozone air pollution. In their evaluation, the per cent of the global population that is exposed to concentrations of PM2.5 above the WHO guideline value of 10 µg m^−3^ annually was 34% in 2010, and would increase to 42% under the CLE scenario, and decrease to 1% under the MFR scenario by 2030, with strong regional differences in the magnitude of the effects. These changes in PM2.5 under the MFR scenario in 2030 could avoid up to 1.5 million premature deaths annually [49].

A multi-model evaluation of future climate and health impacts based on the RCP scenarios showed that by 2030, the range of scenarios resulted in 289 000 premature deaths per year avoided (RCP 4.5) to 17 200 excess premature deaths per year (RCP8.5) for PM2.5 relative to the baseline value from 2000 [34]. Although the baseline years are different, the CLE scenario results from Likhvar *et al*. [49] was similar to the RCP8.5 scenario from Silva *et al*. [34]. By 2100 substantial reductions in PM2.5 result in global premature deaths avoided across all scenarios, ranging from −1.31 million (RCP8.5) to −2.39 million deaths per year (RCP4.5) for the multi-model average [34]. In a follow up study, the premature mortality from changes in air pollution attributable to climate change was isolated from the changes in air pollution attributable to changes in emissions [54]. Changes in air pollution associated mortality owing to climate change are projected to increase under RCP8.5. For PM2.5 the multi-model estimate was 55 600 premature deaths in 2030 and 215 000 premature deaths in 2100. This increase counters the overall global decrease in premature mortality expected under RCP8.5 in 2100 by 16%. For ozone, of the 264 000 (316 000) premature deaths projected by the multi-model average in 2030 (2100) under RCP8.5, an estimated 3340 (43 600) were attributed to the effect of climate change [34,54]. Partanen *et al*. [56] evaluated premature mortality using three RCP scenarios and a high and low aerosol emission scenario based on RCP4.5. Differences in mortality estimates from this study to those provided by Lelieveld *et al*. [50] and Silva *et al*. [34] could be largely explained by differences in baseline mortality rates. The low aerosol emission variant of Partanen *et al*. [56] yielded very similar avoided deaths by 2030 as obtained for MFR in Likhvar *et al*. [49]. Conversely, the authors found by 2030 the highest mortality rate (2 371 800 deaths per year) under the high aerosol emission variant.

**Figure 2.**
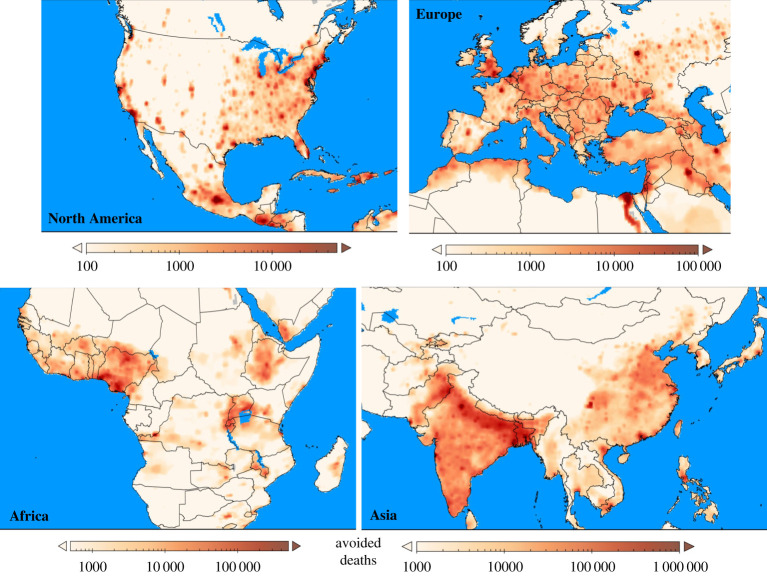
Reduction in annual premature deaths due to PM2.5 and ozone over the period 2020–2100 from co-emissions accompanying accelerated CO_2_ emissions reductions, depicted as regional highlights. Values are all-cause per 0.5° × 0.5° area (approx. 50 km × 50 km at mid-latitudes) without low exposure thresholds. Note different ranges in the panels. Adapted from Shindell *et al*. [57]. (Online version in colour.)

A number of papers evaluated changes based on scenarios linked to the context of the Paris Agreement and the associated 2°C target [53,55,57]. These studies evaluated climate change mitigation scenarios, but also the associated changes in air quality and co-benefits for human health. The two papers by Shindell *et al*. [53,57] used a similar methodology, but one evaluated the effect of US emissions reductions for two scenarios—clean energy and clean transportation—consistent with the 2°C target on global health, while the other investigated the health benefits of accelerated CO_2_ reduction policies globally. Cumulatively from 2020 to 2100, accelerated CO_2_ reductions, would prevent 153 ± 43 million premature deaths (figure 2 [57]). Markandya *et al*. [55] evaluated four scenarios, including scenarios in which stabilization at 2°C and 1.5°C warming were achieved. In all cases, the climate mitigation policies had substantial co-benefits for reducing air pollution emissions and human health. A comparison of the mitigation costs compared to the health co-benefits indicated that the benefits strongly outweighed the costs, with ratios of benefits:costs ranging from 1.4 to 2.45 for the 1.5°C and 2°C stabilization scenarios [55], and even greater by a factor of 5–10 for the US emission reduction scenarios [53]. Vandyck *et al*. [58] evaluated two scenarios, a 2°C stabilization scenario and a Nationally Determined Contributions (NDCs) scenario, documenting that the latter could lead to 71 000–99 000 avoided premature deaths globally in the year 2030 compared to current climate policies. The authors estimated that a more stringent 2°C pathway could avoid 178 000–346 000 premature deaths annually in 2030 and 0.7–1.5 million by 2050, which are similar cumulative health co-benefits as found in the Shindell *et al*. [57] and Markandya *et al*. [55] studies discussed above.

Two papers evaluated sector specific scenarios, both for transportation. Morita *et al*. [51] evaluated three future scenarios, including a reference scenario assuming unconstrained growth and a factor of 4.8 increase in fuel consumed relative to the base year of 2006. The reference scenario resulted in an increase in excess mortality of approximately 5500 deaths from the aviation sector in 2050, relative to approximately 400 excess deaths in 2006 associated with changes in PM2.5. The two improved fuel policy scenarios (technology and operations improvements yielding smaller increases in fuel combustion compared to 2006, and conversion to fully sustainable fuels) still resulted in increased excess deaths in 2050 relative to 2006, but were substantially less than the reference scenario, with approximately 2300 and approximately 1600 excess deaths attributed, respectively [51]. Anenberg *et al*. [52] analysed the health impacts of mitigating excess diesel-related NOx in 11 major vehicle markets. The study found that implementing Euro 6/VI standards where they are not yet adopted, specifically for heavy-duty diesel vehicles, could avoid 104 000 premature deaths in 2040. Beyond Euro 6/VI, further adopting and enforcing next-generation standards that include more stringent real-driving emissions programs would avoid 174 000 premature deaths in 2040 [52]. The differences in premature deaths that could be avoided for these sectors is likely linked to, among other factors, the location of where the emissions occur, with vehicular emissions more prevalent in high population areas.

#### (ii) United States of America

More than 20 papers were published since 2013 that evaluated the health effects of future air quality burdens on human health in the USA (table 1). The majority of the studies examined projections at the national scale, however, a number of studies also focused on certain regions, states or metropolitan areas within the USA. The scenarios evaluated ranged from the globally relevant RCPs to those that specifically considered US regional energy policies.

Kim *et al*. [71] investigated the changes in ozone-related mortality in the USA between 2001–2004 and 2057–2059 under a low-to-medium emission scenario, RCP4.5, and a fossil fuel intensive emission scenario, RCP8.5. They also evaluated the sensitivity of the mortality estimates to the input variables. To derive excess mortality due to ozone, Kim *et al*. [71] based concentration-response functions (CRFs) on the association between non-accidental, all-cause mortality and short-term exposure to maximum daily 8-h average (MDA8) O_3_ (following [98]) and maximum daily 1-h average (MDA1) O_3_ (following [98,99]) and calculated excess mortality on county level. Their results showed, compared to the base period (averaged across two different CRF coefficients, and four population scenarios), approximately 1300 additional premature deaths under RCP8.5, while approximately 2100 premature deaths could be annually avoided under RCP4.5, with significant variation spatially. The largest amount of uncertainty in the estimates was associated with the RCP emission pathways [71].

Martinich & Crimmins [87] also estimated premature mortality associated with health effects of surface ozone based on RCP4.5 and RCP8.5 projections. This was part of a broader evaluation of projections of climate damages and adaptation potential across diverse sectors of the USA for a broad range of endpoints, not only human health, but also infrastructure, agriculture and ecosystems. All other effects were, however, limited to changes in climate and not air quality and no adaptation measures were included in the sectoral impact analysis for air quality. Five hundred and fifty excess deaths annually were estimated under RCP4.5 and 790 excess deaths annually under RCP8.5 in 2050, with these values increasing to 1200 and 1700 excess deaths annually, respectively, in 2090 [87]. In comparison to the Kim *et al*. [71] study, these values were similar for RCP8.5 but do not show the same avoidance of excess deaths under the RCP4.5 scenario.

A novel study by Abel *et al*. [82] evaluated the air quality related health outcomes due to heat-driven adaptation of increased air conditioning demand for buildings for the Eastern USA. The study compared representative present-day (2011) and mid-century (2069) climate scenarios (based on the IPCC A2 scenario) with and without exacerbated power sector emissions from adaptation in building energy use from increased air conditioning. The impact of climate change alone resulted in a projected increase in summer air pollution related premature mortality of roughly 13 000 deaths due to PM2.5 and 3000 deaths due to O_3_. Air conditioning adaptation accounted for 645 and 315 of these PM and O_3_ related annual excess deaths, respectively [82]. The role of such adaptation measures and feedback effects is not quantified in most studies. This shows, however, that such adaptation measures could have substantial impacts on future projections related to air quality and health impacts.

In summary, the recent USA focused literature shows diverse futures for health burdens attributable to PM or ozone. While ozone health burdens are generally projected to increase under business as usual or planned legislation scenarios, reduced adverse health effects emerge regionally (particularly the Eastern USA) under scenarios considering more stringent precursor emission controls. Under RCP8.5 ozone increases are projected for the Western USA, while decreasing ozone burdens are projected for the Eastern USA [100]. For PM, future projections indicate reduced adverse health effects from emission reductions, but additional adverse health effects from population growth, with larger populations being exposed to unhealthy PM levels at the regional level. Studies considering health effects of both ozone and PM generally indicate health benefits are dominated by reductions in PM.

#### (iii) Asia and Australia

A few more than 10 studies explicitly evaluated the health effects of future air quality burdens for Asia and Australia. Almost all studies focused on single countries, and in some cases, urban areas. The majority of the studies conducted projections for China.

The studies by Qin *et al*. [62] and Yang *et al*. [63] investigated future air quality and health benefits for specific policy developments in China using the ECLIPSE_V5a_CLE reference scenario as a baseline. Qin *et al*. [62] evaluated the implications of China's synthetic natural gas (SNG) development strategy for 2020, whereby all planned production of SNG is deployed to replace coal in the power, industrial or residential sectors. Yang *et al*. [63] investigated the effect of fulfilling the 400GW national photovoltaics (PVs) capacity in 2030 as per the China Renewable Energy Roadmap 2050, which is intended to reduce emissions from coal fired power plants. The results vary depending where the PV capacity is installed and thereby the type of power plants being replaced, as well as the amount of inter-provincial PV electricity transmission. The benefit from the SNG scenarios was greatest if deployed to replace coal use in the residential sector, with approximately 32 000 premature deaths avoided annually in 2020 from outdoor air pollution alone. If indoor air pollution was included, values would be much higher. If the SNG were deployed to replace coal use in the industrial or power sectors, the air pollution associated avoided premature deaths would be much lower, 3100 or 560 annually, respectively, nationwide in China in 2020 [62]. The results from the PV scenarios showed that installation in eastern China with inter-provincial transmission has the largest benefits as it displaces the dirtiest coal power plants. The projections estimated a reduction in premature deaths associated with PM2.5 air pollution of 10 000 by 2030 relative to the base case [63]. Both studies explored the health effects of replacing coal-based energy with alternative energy forms in China, and agreed that energy transition is most promising in the residential sector. Qin *et al*. [62] reported larger health gains for 2020 than Yang *et al*. [63] for 2030. This difference can be explained by the full regional consideration of China's coal-based SNG development plan by Qin *et al*. [62], while Yang *et al*. [63] compared the efficiency of different regional solar PV deployment and utilization scenarios and did not quantify effects of full PV deployment.

**Figure 3.**
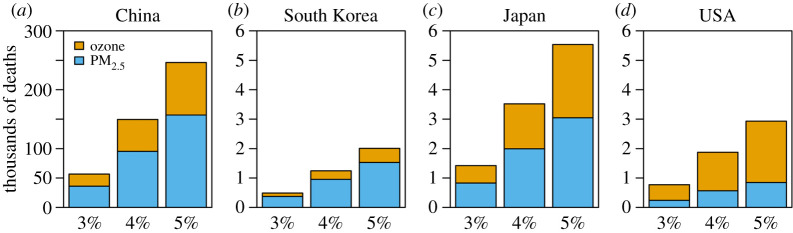
Avoided PM2.5- and ozone-related premature deaths under three climate policy scenarios relative to No Policy in China (*a*) and three downwind countries (*b*)–(*d*) in 2030. Ozone-related deaths are calculated using CRF in Turner *et al.* [101]. Note different scale for panels (*b*)–(*d*). From Li *et al*. [69]. (Online version in colour.)

A recent study evaluated air quality and health co-benefits of China's climate policy for PM2.5 and ozone nationally and for three populous countries downwind of China (South Korea, Japan and the USA) [69]. The authors considered policy scenarios with CO_2_ reductions of 3%, 4% or 5% per year between 2015 and 2030. All scenarios yielded substantial health gains due to associated PM2.5 and ozone reductions, with the amount of net gain related to the ambition in CO_2_ reductions (figure 3). For example, Li *et al*. [69] reported detailed health gains for the 4% CO_2_ reduction path. Here, the largest effects emerged with 95 200 PM2.5-related and 54 300 ozone-related avoided premature deaths locally in China relative to the base year. The study also found substantial health benefits for downwind countries with 600 (the USA) to 2000 (Japan) PM2.5-related and 300 (South Korea) to 1500 (Japan) ozone-related avoided premature deaths in 2030. Generally across all CO_2_ reduction scenarios benefits due to reduced PM2.5 were larger than those for ozone, and cumulative health benefits in China exceed those in downwind countries by about a factor of 50–100 (figure 3).

**Figure 4.**
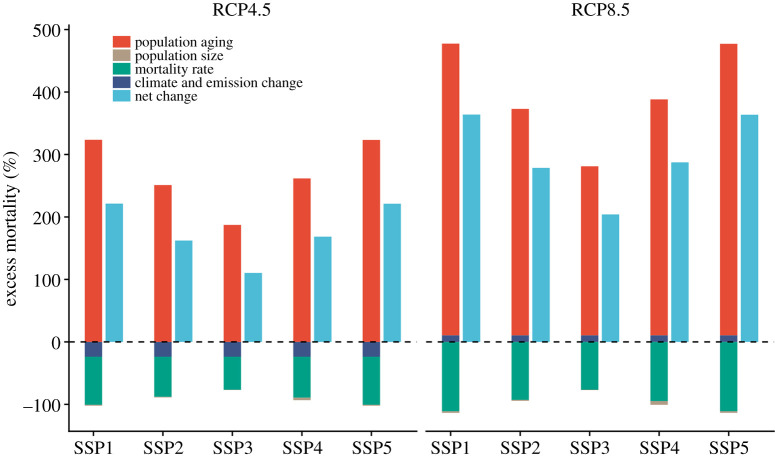
Changes in ozone-related mortality according to climate and population changes from 2013 ± 2015 to 2053 ± 2055. Population changes include both population size changes and population ageing. Mortality rate indicates age-group-specific baseline mortality rate changes. Future changes (%) of annual ozone-related mortality for the population aged 5 years and above in 2053 ± 2055 were calculated relative to the historical period 2013 ± 2015. RCP4.5 and RCP8.5 represent moderate and high global warming and emission scenarios, respectively. SSP1 ± 5 represent five population change scenarios under different shared socioeconomic pathways. From Chen *et al*. [65]. (Online version in colour.)

A study of 104 cities across China used a combination of the RCPs (RCP4.5 and RCP8.5) and six population change scenarios from the SSPs, to evaluate not only excess ozone-related future mortality from changes in emissions and climate, but also how the ozone-related excess mortality rates changed when considering and adjusting for population change and ageing [65]. Compared to the estimates for the historical time period (2013–2015) for which roughly 13 900 deaths were attributed to short-term exposure to ambient ozone air pollution in the 104 Chinese cities, a decrease in mortality (approx. −3300 deaths) was observed for the RCP4.5 scenario annually in 2053–2055, and increase observed under RCP8.5 (approx. 1500 deaths annually), considering no population change. Using age-group-specific concentration-response functions and baseline mortality, rather than all-age values, increased the historical mortality estimate to approximately 21 600 deaths. This indicates the significant influence such choices can have on the magnitude of the derived mortality estimates. Population ageing emerged as such an important factor that it offset the decrease in excess deaths projected under RCP4.5 and dominated the net increase in excess deaths under RCP8.5, despite projected decreases in overall population size and expected decreases in age-group-specific mortality rates, as in figure 4 [65]. Changes in population demographics, and specifically ageing, are not factors that are typically considered in many of the studies investigating changes in the effects of future air quality. This study, however, highlights the important role such changes may play, likely resulting in substantial underestimates of future air pollution related excess mortality.

Overall, the studies evaluating changes in Asia and Australia show that the projected changes in health effects are strongly dependent on the level of ambition, with scenarios that follow currently planned legislation and/or business as usual scenarios projecting an increase in adverse health effects, while reductions in adverse health effects are observed for scenarios and policies that require more ambitious emission reductions or greater technology changes. Furthermore, a number of the studies agree that there will be a substantial climate penalty for ozone-related adverse health effects, requiring greater reductions to offset the effects of climate on ozone concentrations, with the largest impact in areas with the highest population densities and ozone concentrations.

#### (iv) Europe

In addition to the global studies that include a regional investigation for Europe, over the last 6 years three studies have focused on the effects of future air quality on the health of the European domain. All studies included both ozone and PM, while one study specifically isolated the effect of climate change on future air quality associated health impacts [91].

**Figure 5.**
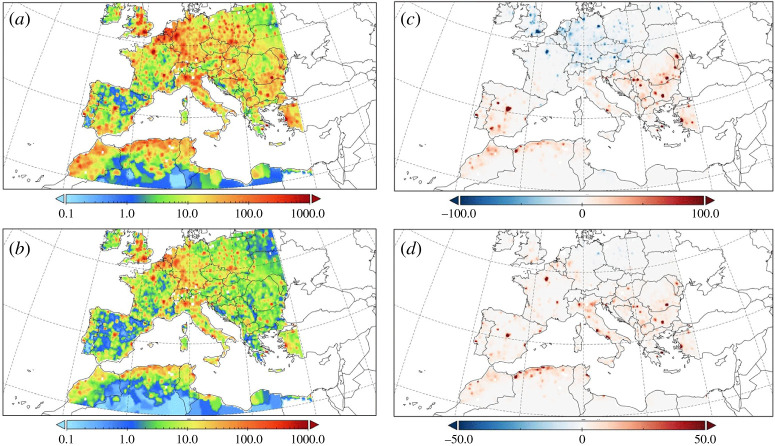
(*a*) Present cases of premature deaths (PD) and (*b*) associated costs, in millions of euros. (*c*) Changes projected in PD and (*d*) changes in costs (millions of euros) under the RCP8.5 scenario (2071–2100). From Tarín-Carrasco *et al*. [91]. (Online version in colour.)

Tarín-Carrasco *et al*. [91] isolated the effects of climate change on air-pollution-related-pathologies in central and southern Europe by holding emission levels and population density constant at present-day (1996–2015) levels while considering future climatic conditions under RCP8.5 (2071–2100). The results showed an increase in premature deaths, attributable to climate driven air quality degradation of 94 900 cases annually (valued at an additional EUR 27 billion in external costs) (figure 5). This represents a 17% increase compared to present day air quality-related premature mortality (418 700 cases; EUR 158 billion in external costs).

Schucht *et al*. [89] investigated two Global Energy Assessment (GEA) scenarios, a reference scenario with no climate policy (consistent with RCP8.5) and a climate mitigation scenario limiting global temperature increase to 2°C by the end of the twenty-first century (consistent with RCP2.6). Both scenarios, however, include an updated representation for air pollutant emissions considering all currently legislated air quality policies until 2030. The results showed that air quality policies alone (no climate policy) effectively reduce the adverse health impacts associated with PM2.5, while the ozone-related adverse health impacts are much more dependent on climate policy [89]. Specifically, premature deaths from acute exposure to ozone, estimated to be 31 000 in 2005, were projected to increase to 48 000 in 2050 for the scenario with no climate policy (with the increase being largely attributed to a growing and ageing population in Europe over this time period). Under the climate mitigation scenario, the premature deaths from acute exposure to ozone across Europe were reduced to 7000 in 2050. In comparison, the health impacts of PM2.5 in these scenarios showed further improvements due to climate policy. From 2005 to 2050, the number of years of life lost (YOLL) already decreased from over 4.6 million to 1 million following the implementation of the air quality policy even in the absence of any climate policy. The additional implementation of an ambitious climate policy further reduced the YOLL to 0.3 million in 2050 [89].

Overall, the results from the European studies agree well with those for the USA or Asia and Australia. Recent research indicates larger health benefits from reductions in PM than ozone. Health benefits emerge on local and regional scale following more stringent air pollution policies but penalties also emerge, particularly for ozone, in the absence of effective climate policies.

#### (v) Limitations

For simulations focusing on air quality impacts no community standard or strategic protocol exists, as has been developed e.g. for climate change simulations developed within the Coupled Model Intercomparison Project or simulations focusing on chemistry–climate connections developed within the Atmospheric Chemistry and Climate Model Intercomparison Project (ACCMIP) or Chemistry Climate Model Initiative (CCMI). The available studies vary strongly in the design of the modelling chain and the scenarios, regions, time periods and mortality and morbidity measures considered. The diversity of approaches and scenarios can be viewed as a strength in understanding the scope of possible future effects, as well as identifying consistency in projected effects. However, coordination of quantified effect metrics, as well as transparency in model assumptions and treatment of processes would support comparisons. Thus a number of important considerations and limitations can be identified across the studies included here regardless of global or regional focus. A selection of these factors has been summarized in table 2.

**Table 2.** Selected factors that are limitations or influence the outcome of model studies on future projections of air pollution effects on human health.

limitations
scenario type (global scenarios, e.g. RCPs versus local policy)
multi- versus single-model analyses, lack of multi-member ensembles
representation of atmospheric processes, feedbacks, chemistry–climate interactions, natural emissions
model biases in pollutant concentrations and distributions
baseline (year, conditions, assumptions)
future time span simulated
length of time period simulated (number of years, individual year or select month(s) or season(s))
CRFs used for quantification of health impacts, as well as the spatial and temporal resolution of the population dataset used in the concentration-response functions
metrics quantified (and base year or period)
consideration of air pollution and temperature interactions
consideration of population and demographic change; limitations in age range considered
consideration of changes in population vulnerabilities, urbanization, healthcare, air pollution composition

For one, as is demonstrated by those publications that are multi-model studies, the underlying representation of atmospheric processes, chemistry–climate interactions and treatment of natural emissions in the different model set-ups can result in large differences for the same scenario. For example, Silva *et al*. [34] found that for most cases in the multi-model study, uncertainty in modelled air pollutant concentrations was the greatest contributor to uncertainty in mortality estimates. Health impact studies are based on CRFs and the choice of these functions for estimating air pollution associated mortality or morbidity can have a substantial impact/lead to great uncertainty [90], as well as assumptions about the toxicity of particles [50]. A recent meta-analysis combining multiple appropriate epidemiological studies offers a first possible solution to this end [102].

Due to natural variability, the years simulated will influence the outcome of the study results. A review paper focused on the climate change penalty for ozone air quality found that for model projections, a minimum of 15-year averaging windows are required to smooth out the ‘noise’ due to natural climate variabilities and to distinguish signals forced by anthropogenic climate warming [13], while another study found that the precise number of simulation years required for a given degree of accuracy will depend on the timing and strength of the policy in question [85].

Changes in temperature and oxidative capacity will influence secondary organic aerosol, but the physical-chemistry processes are not well understood and therefore any future effects are generally not included in modelling studies [90]. In addition, few studies take interactions between temperature and air pollution on health effects into account, but recently studies have emerged that show, generally, air pollution related mortality is greater for higher temperature or warmer days [10,102]. Changes in atmospheric stagnation events, precipitation and heat waves will affect future concentrations of PM and ozone. While changes in stagnation will effect both PM and ozone, heat waves have a larger effect on ozone concentrations and changes in precipitation frequency and amount will have a larger influence on PM. Taken together these climate extremes may represent an important mechanism through which climate change will affect future air quality [8–11].

Many studies acknowledge that changes in population totals and demographics will substantially affect mortality estimates (see also the summary of Chen *et al*. [65] included above), but do not include such changes in the projections conducted. For those studies that have included such changes in the projections, consideration of these factors typically shows substantial increases in mortality (e.g. [65,68,90]). In addition, using future baseline mortality rate projections, rather than maintaining constant conditions will also influence the magnitude of the results, generally reducing the magnitude of the benefits [49]. Including only a limited age range, e.g. no mortality included for people under 25, will also underestimate mortality. In addition, related factors such as changes in healthcare system(s), population vulnerabilities, increasing urbanization and/or changes in the composition of the future air pollution mix are generally not being considered but would influence projections [49,103]. Finally, imbedded in some of these factors is the differential impacts of air pollution on demographic groups, with lower socioeconomic conditions generally linked to higher air pollution exposures [104]. Changes in socioeconomic conditions and the differential effects on various demographic groups are also not being considered in these studies and will likely continue to evolve over time and influence projections. An important consideration in future air quality management would be to minimize differential exposure of different socioeconomic populations to air pollutants.

### (b) Crops

Compared to the impacts on human health, fewer recent papers investigated the impacts of future air quality on crop production. Most crop studies are global in scope and presented additional regional or country-level insights. These works focused largely on the major crops of the developed world (maize, rice, soya bean and wheat) but vary in future scenario, quantification metric and process complexity. An overview of the studies presented here is listed in table 3. The description and discussion below is organized by study type and topic and generally proceeds from more well-studied to more uncertain.

**Table 3.** Studies on future changes in air quality effects on crops. RY, relative yield.

publication	region(s)	scenario(s)	base year	future year(s)	ozone	PM	crop(s)	crop units	notes
Van Dingenen *et al*. [105]	Global; selected country values	current legislation (CLE)	2000	2030	X		maize, rice, soya bean, wheat	RY loss	monetary valuation included
Averny *et al*. [106]	Global; selected country values	A2, B1	2000	2030	X		maize, soya bean, wheat	RY loss	monetary valuation included
Shindell *et al*. [107]	Global; selected country/regional values	tight on-road vehicle emissions	2000	2030	X		maize, rice, soya bean, wheat	RY loss, production totals	monetary valuation included
Amin *et al*. [108]	East Asia	emissions reduction policy success, reference, failure	1980	2020	X		maize, rice, soya bean, wheat	RY loss	monetary valuation included
Tai *et al*. [109]	Global; selected country/regional values	RCP4.5, RCP8.5	2000	2050	X		maize, rice, soya bean, wheat	relative production loss	crops equated using calorie-equivalence, climate impacts included
Chuwah *et al*. [110]	Global; regional values	RCP2.6 type, RCP6.0 type (low + high)	2005	2050	X		maize, rice	RY loss	land use impacts included
Capps *et al*. [111]	USA	US Clean Power Plan options		2020	X		cotton, maize, potato, soya bean	potential productivity loss (PPL)	
Tai & Val Martin [112]	USA, Europe; extension to China, India values	RCP4.5, RCP8.5	2000	2050	X		maize, soya bean, wheat	relative production loss	uses partial derivative-linear regression (PDLR), climate impacts included
Schiferl & Heald [113]	Global; selected country/regional values	RCP4.5, RCP8.5	2010	2050	X	X	maize, rice, wheat	relative production loss	
Vandyck *et al*. [58]	Global; selected regional values	only current climate change policies, NCDs, 2° target, BAT, SLE, FLE	2010	2030, 2050	X		maize, rice, soya bean, wheat, various aggregates	RY loss	
Miranda *et al*. [114]	Portugal	RCP8.5	2000	2100	X		wine grapes	productivity, quality	climate impacts included

The impacts of air quality on crops are most frequently described in terms of relative yield (RY), which refers to the area-normalized production (yield) given a certain amount of pollutant compared to the yield in the absence of that pollutant entirely or below a certain threshold. The change in RY caused by that pollutant is then described as the RY loss or gain. When describing impacts on a global or regional scale, total or relative production values can be used to account for the land area used for each crop. Several studies further translate impacts on crop yield/production into unifying units such as commodity pricing or calorie content, although this can add additional uncertainty to future projections.

**Figure 6.**
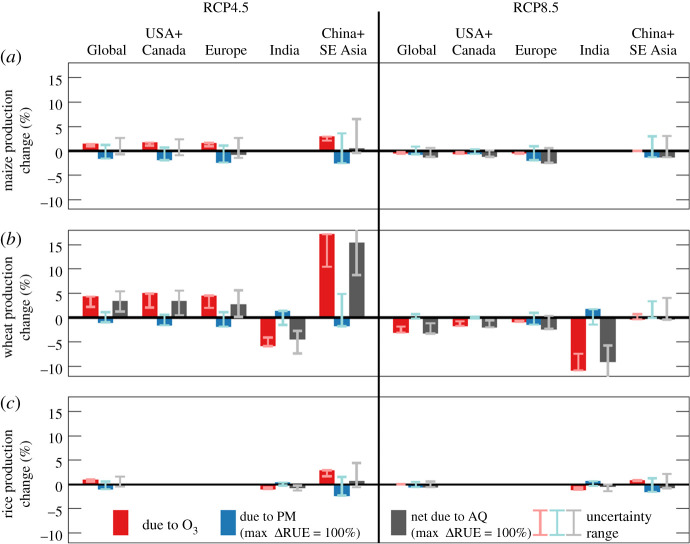
For both RCP4.5 (left) and RCP8.5 (right) emissions scenarios: regional relative change in crop production due to surface ozone (red bars/leftmost bars in clusters), PM with maximum diffuse effect (blue bars/middle bars in clusters), and both ozone and PM (grey bars/rightmost bars in clusters). Change from 2010 to 2050 for (*a*) maize, (*b*) wheat and (*c*) rice. Error bars indicate range of production from 0 to –10 ppb surface ozone concentration correction, from minimum to maximum diffuse PM effect, and from both effects, respectively. Regions with a base production lower than 5% of the global total are not shown. Relative change calculated from 2010 base production. From Schiferl & Heald [113]. (Online version in colour.)

The largest body of literature focuses on the decrease in crop yield associated with ozone damage using CRFs. In these studies, future scenarios are for precursor emissions only with constant future meteorology. This isolates the impact of possible future emissions regulations, rather than confounding with additionally uncertain climate projections. Van Dingenen *et al*. [105] found global RY loss for 2000 due to ozone to be 3–5% for maize, 3–4% for rice, 6–16% for soya beans and 7–12% for wheat. Under a CLE scenario for 2030, additional losses were projected to be 1–2% for rice and 2–6% for wheat, with less than 1% change for both maize and soya beans. This response translates into an additional loss of $14–26 billion. Ranges here reflect different available ozone exposure metrics used in CR functions. Using the same approach, Shindell *et al*. [107] found similar, but slightly lower, present day global RY loss for all four crops. In their analysis, imposition of ‘tight-controls’ for on-road vehicle emissions in 2030 resulted in a decrease in global wheat RY loss of 1.1% and savings of 6.1–19.7 million metric tons of crop losses worth over $1.5 billion. The A2 (high emission) scenario employed by Averny *et al*. [106] showed an additional global RY loss of 2–3% for maize, 1–11% for soya bean and 2–10% for wheat by 2030 compared to 2000, with the B1 (low emission) scenario contributing to less RY loss overall, a maximum 2% additional loss for wheat. The value of additional crop losses in 2030 was found to be $6–17 billion for A2 emissions and only $1–3 billion for B1 emissions. More recently, implementation of the RCP scenarios found that changes in global ozone-related crop (maize, rice, soya bean and wheat) damage led to a production gain of 3.1% (0.22 × 10^15^ kcal) for RCP4.5 and production loss of 3.6% (0.26 × 10^15^ kcal) for RCP8.5 by 2050 compared to 2000 [109]. Similarly, Schiferl & Heald [113] saw a global increase (decrease) in production of 1.4% (0.5%), 1.0% (0.1%) and 4.4% (3.0%) for maize, rice and wheat, respectively, under RCP4.5 (RCP8.5) in 2050 compared to 2010 due to the projected crop area- and growing season-weighted decrease (increase) in ozone concentrations (figure 6). Considering recent policy implications, Vandyck *et al*. [58] found that more stringent emissions goals limiting warming to 2°C could double the gains in global crop yields made by reducing ozone concentrations compared to those limits set by NDCs. This limited emissions policy would increase maize, rice, soya bean and wheat productivity by 0.8–1.5%, 0.2–0.8%, 1.8–2.7% and 0.9–1.7%, respectively, in 2030 compared to a reference case without climate change mitigation.

While the projected increase in ozone damage on global total crop production was estimated to be less than approximately 10% by 2030/2050, and there is the potential for global total loss reduction under more restrictive emissions scenarios, these projections vary strongly on the national and regional level. In East Asia, Amin *et al*. [108] evaluated a ‘policy failure’ case (higher emissions) which resulted in an increase in RY loss due to ozone of 9–10% for maize, 6–12% for rice, 13–33% for soya bean and 5–27% for wheat in 2020 compared to a baseline in 2000 (which is 17–35% RY loss for wheat). This case increased value lost from ozone damage from $14 billion in 2000 to $27 billion in 2020 in this region. Using an RCP6.0-type high emissions scenario, Chuwah *et al*. [110] estimated about 10% increase in RY loss in maize and rice between 2005 and 2050 in the Middle East, India and China. With more stringent policy controls, RY loss for these crops would decrease by 2050 throughout the USA, Europe and Middle East, but would remain significant (greater than 15%) in parts of China and India. Such regional differences are consistent with results found by Schiferl & Heald [113] (figure 6). Under the RCP8.5 emission scenario, wheat production is expected to decrease by 10% in India between 2010 and 2050 due to ozone damage but less than 3% in the USA + Canada, Europe, and China + South East (SE) Asia regions. Emission controls on ozone in the RCP4.5 scenario increased wheat production in China + SE Asia by over 15% and nearly 5% in USA + Canada and Europe, while India's production loss was reduced by about 5% in 2050 compared to 2010. In the USA, Capps *et al*. [111] implemented versions of the Clean Power Plan and found that such regulations would reduce potential productivity loss (PPL) due to ozone by up to 16% for maize (although with very low reference PPL) and 8% for soya bean in 2020 compared to a reference case. Extending the RCP8.5 pathway to 2100 for Portugal, Miranda *et al*. [114] suggested approximately 15% increase in productivity and quality for wine grapes due to emissions reductions, compared to approximately 35% decrease that would be expected under constant current emissions.

**Figure 7.**
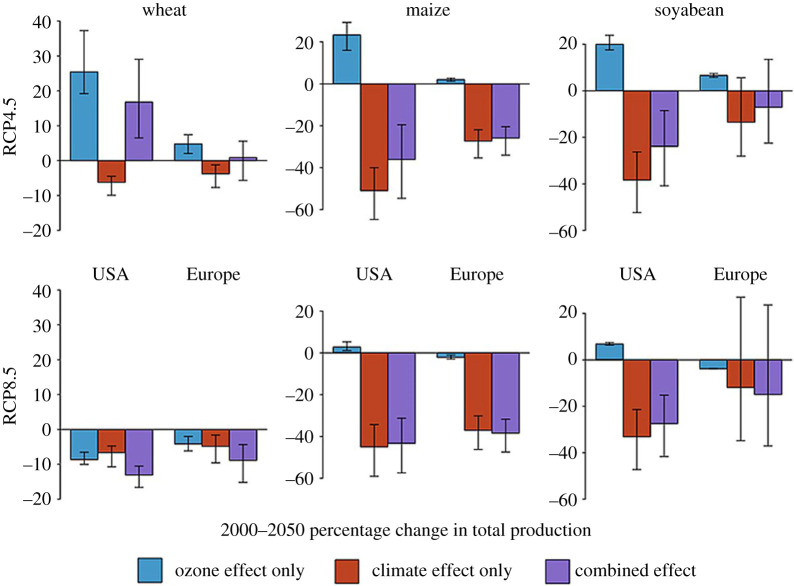
Projected 2000–2050 percentage changes in total production for wheat, maize and soya bean for the USA and Europe following RCP4.5 and RCP8.5 scenarios under individual (blue and red/left and middle bars) and combined (purple/right bars) effects of ozone pollution and warming. Bars indicate the mean changes and the notches indicate the 90% confidence intervals as estimated from Monte Carlo method, denoting a ‘very likely’ change if the interval does not overlap with zero. From Tai & Val Martin [112]. (Online version in colour.)

Tai & Val Martin [112] derived new relationships using partial derivative-linear regression (PDLR) for the impact of ozone on crops using observed crop yield data and ambient ozone measurements in the USA and Europe. This method allowed for spatial differentiation in the response of crops, unlike the CR equations, which are usually applied broadly to all areas. Their work highlights the possible development of stronger ozone tolerance by crops in ozone-stressed areas, with crop-producing areas away from ozone hotspots more sensitive to ozone stress than those located near urban regions. Using the PDLR relationships, RCP4.5 emission reductions in 2050 led to a 25% increase in US wheat production compared to 2000 and only 4% increase in Europe (figure 7). Since US production areas are more remote than those in Europe, this distinction is larger than that found by Tai *et al*. [109] using uniform CR equations. Applying these PDLR relationships (from USA and Europe) to India and China using RCP8.5 emissions for 2050 compared to 2000 showed ozone may have a larger impact than found when using the CR equations. While these impacts were quantified, the relationship is better qualified, as the authors stress that the uncertainty in cross-continental application is large.

Little work has been done to quantify the future impacts of PM on crops. Through scattering, PM decreases the total, but increases the diffuse portion of light intercepted by a plant. This diffuse light has an enhancing effect by more evenly distributing light photons throughout the plant leaf canopy. Indeed, PM can be beneficial to plants by increasing their light use efficiency, if not offset by the negative effects of the reduction in total light. Therefore, unlike ozone, PM can cause either a net increase or net decrease in crop yield/production depending on the PM concentration and composition in the atmosphere. Using the maximum impact from various uncertain relationships, Schiferl & Heald [113] found changes in PM (largely reductions) could cause a global decrease in production of 1–2% for maize, rice and wheat under RCP4.5 in 2050 compared to 2010, with less impact for RCP8.5 (figure 6). The authors pointed out the need for more physiological studies to establish the current impacts of PM, which could be important as particles are removed from the atmosphere and reduce the net enhancing impact on crops. This was especially clear in the RCP4.5 scenario, where nearly 3% of maize and rice production could be lost in China + SE Asia due to lower PM levels. In this case, the PM effects have the potential to offset improvements from ozone reduction. By contrast, higher levels of PM in India under RCP8.5 could lead to an increase in wheat production, but this increase would be overwritten by the large loss in production due to ozone.

Overall, there is consensus that ozone damage has a significant negative impact on global crop production. Future projections indicate that this damage will be lessened and crop production enhanced in areas that implement ozone-reducing policies, which seems most likely to be outside of Asia. Future reductions in PM through air quality controls may have a slight negative impact on crop production, but this is highly uncertain and motivates further study.

#### (i) Limitations

The higher temperatures associated with a warmer climate both increase ozone production and directly impact the productivity of crops. For example, studies comparing temperature effects to ozone damage indicate future crop production could be dominated by the negative impacts of higher temperatures, but air quality policy offers a significant opportunity for mitigation [109,112]. This pattern is especially relevant for more restrictive emissions scenarios such as RCP4.5 (figure 7). PM also affects cloud formation and net radiation reaching the surface, which in turn impacts significant factors for crops including hydrology and temperature. In the event of future geoengineering, where PM is introduced to the stratosphere, a study by Proctor *et al*. [115] shows this would have little net impact on crops in 2050, where the increase in global crop yield due to lower temperatures is largely offset by the decrease in available light. These interactions are not considered in this review, but are important for a more holistic understanding of the impacts of future climate and air quality on global crop production.

The studies in this review also do not take into account future changes in land use (e.g. conversion of forest into cropland), the distribution of crops (e.g. substitution for ozone- or drought-resistant varieties) or planting/harvesting calendars (e.g. longer summers) as development advances and the climate changes. Although atmospheric deposition of nitrogen and sulfur can have positive impacts on crop production as essential limiting nutrients to plants [116,117], studies do not quantify their impacts on future crop production. Therefore, policies regarding water and nutrient availability (e.g. those that reduce the atmospheric burden of nitrogen and sulfur) are important to consider for additional study with regards to changing food production in the future. These policies may impact the ability of crop production to reach its full potential when enabled by crop-enhancing air quality policy, such as the reduction in ozone pollution, as supplemental application can be prohibitively costly or be environmentally impactful in other ways.

Finally, studies of future air quality impacts on crop production largely focus on the crops of the Northern Hemisphere. However, the air quality in Africa is expected to decline in combination with projected population growth and increased combustion emissions [118,119], and there is little mention in current studies of the impacts of air quality on the dominant crops (e.g. sorghum, millet, pulses) of this region. Additional studies of these crops would help to understand to what extent future ozone and PM levels could significantly impact Africa's already vulnerable food system.

### (c) Ecosystems

Effects of air pollution on ecosystems have been studied and established for decades. These effects are manifested through a host of drivers such as impaired visibility from PM, phytotoxicity due to elevated O_3_, soil and freshwater acidification of forest and aquatic ecosystems associated with atmospheric deposition of acid and acidifying compounds (SO_2_, NO_x_, NH_3_) [120,121], eutrophication of terrestrial and marine ecosystems associated with atmospheric deposition of nitrogen (oxidized and reduced nitrogen) [122,123], and contamination of terrestrial, freshwater and marine ecosystems due to atmospheric deposition of mercury [124] (figure 1). With the exception of visibility and plant effects of ozone, which occur over the growing season cycle, ecosystem effects (acidification, eutrophication, mercury contamination) generally are the result of atmospheric deposition. Atmospheric deposition occurs as wet deposition, which includes rain, snow, sleet or hail; as dry deposition, which includes atmospheric particles or gases; or as cloud or fog deposition, which is more common at high elevations and in coastal areas. Wet deposition is fairly well characterized by monitoring at more than 270 National Atmospheric Deposition Programs (NADP; http://nadp.sws.uiuc.edu) in the USA, 135 sites in European Monitoring and Evaluation Programme (EMEP; www.emep.int), National Acid Rain Programs and Routine Monitoring Network on Acid Rain in China and Acid Deposition Monitoring Network in East Asia (EANET; www.eanet.asia) at 54 sites in 13 participating countries in Asia, and Global Atmospheric Watch by the World Meteorology Organization (WMO-GAW). Although wet deposition is relatively intensively monitored, it can be highly variable spatially. Dry deposition involves the deposition of gaseous and aerosol species and is less intensively monitored than wet deposition. It is highly dependent on topography, meteorological conditions and vegetation characteristics, which can vary markedly over short distances in complex terrains [125,126]. As a result, dry deposition is poorly characterized and highly uncertain.

Meteorological conditions and climate change affect the quantity and distribution of wet and dry deposition. Wet deposition can occur as removal within a cloud known as ‘rainout’ or scavenging from the atmosphere by precipitation called ‘washout’ [127]. Changes in the quantity and the intensity of precipitation will alter the quantity of wet deposition and the distribution between wet and dry deposition. Changes in air temperature and air and soil moisture will alter emissions, partitioning and deposition of gases and particulate matter and therefore dry deposition. Due to high leaf area index, changes in forest canopy structure, exposure and function can greatly influence dry deposition. Coniferous vegetation is more effective than hardwood vegetation in scavenging air pollutants from the atmosphere (e.g. [128]) so shifts in vegetation type alter dry deposition. Changes in canopy biomass and stomatal conductance associated with meteorological conditions or atmospheric CO_2_ will also alter dry deposition [129].

The time scale of changes in atmospheric deposition is typically on the order of many decades to a century [47], except for extreme events. As a result, the changes in plant and soil processes within watersheds in response to atmospheric deposition occur over long time scales, again typically many decades to centuries. These long-term changes are largely due to relatively large soil pools which innately buffer soil solutions and mediate hydrologic and biogeochemical processes within watersheds. The extent of soil pools vary with soil mineralogy, depth and age as well as climate and vegetation. For example, inputs of sulfur, nitrogen and mercury to watersheds are largely retained in soil before export from the watershed. For acidification, inputs of strong acids (sulfuric, nitric acid) deplete soil pools of available nutrient cations (calcium, magnesium) or mobilize dissolved inorganic aluminium, which is toxic to plants and fish. The long time scale of ecosystem response to air pollution is in stark contrast to the relatively rapid response of human health, visibility or crops to changes in ambient air quality. These long time scale changes in atmospheric deposition and associated ecosystem processes are consistent with the long time scales of changes in meteorology, hydrology, and plant soil and microbial processes that occur in watersheds in response to climate variability and change [130,131]). Because watershed losses for elements largely occur by fluvial processes or land-atmosphere exchange and because microbial and plant processes are highly sensitive to temperature and soil moisture, ecosystem impacts of air pollution are closely coupled with meteorological and therefore climatic conditions.

Because of the complexity of ecosystem response to air pollution and the different responses for different air pollutants in different ecosystem types, we give three case studies to illustrate the interplay between climate and air pollution effects. The first example is a contemporary observation involving effects of acid rain and tree health and demonstrates the complexity of capturing interactions between air quality and climate with organism response in future projections. In the 1990s, it was demonstrated that acid deposition was impairing the health of red spruce (*Picea rubens*). Red spruce is generally found in montane areas and is native to eastern North America, ranging from eastern Quebec and Nova Scotia, west to the Adirondack Mountains and south through New England along the Appalachians to western North Carolina. The mechanism affecting tree health was the loss of membrane-bound calcium from needles from elevated leaching by acid deposition, which decreased the cold tolerance of red spruce [132]. Under this condition cold winter temperatures caused damage to the current year needles. If needle damage and loss occurs over multiple years it results in the mortality of trees. In recent years, damage to red spruce has been almost completely mitigated due to decreases in acid deposition associated with air quality management in North America, coupled with marked decreases in very cold winter temperatures due to climate change [133].

The second example involves climate change effects on mercury. Although there are sites of localized industrial contamination, most ecosystems derive mercury inputs from atmospheric deposition [124]. As mercury is a global pollutant, virtually all ecosystems are subject to mercury contamination by atmospheric deposition. Within reducing environments (e.g. wetlands, sediments) inputs of mercury can be converted to monomethylmercury, which is readily bioaccumulated and biomagnified through food chains. Elevated exposure to humans and wildlife (songbirds, fish piscivorous birds and mammals) typically occurs through consumption of foods elevated in methylmercury, which is a neurotoxin that can affect a number of health endpoints [134–136]. All components of the transport and processing of mercury in the environment, atmospheric transport and deposition, evasion, terrestrial and aquatic processing, methylation, and trophic transfer, are affected by climatic and hydrological conditions and therefore are strongly impacted by climate change [129].

For many developed countries, the dominant human exposure to mercury occurs by consumption of marine fish with elevated concentrations of methylmercury [137]. In marine food chains, methylmercury can bioaccumulate by a factor to 10–100 million in top predators. Schartup *et al*. [27] used a food web bioaccumulation model to examine the mechanisms driving temporal trends in concentrations of methyl mercury in Atlantic bluefin tuna (*Thunnus thynnus*) in the Gulf of Maine. They found that recent and projected future increases in the methylmercury concentrations in Atlantic bluefin tuna were driven by increases in sea surface temperature rather than changes in mercury emissions and deposition. Climate change is likely to aggravate human exposure to methylmercury through marine fisheries, suggesting that more aggressive controls of mercury emissions and releases will be needed to protect ecosystem services and human health.

**Figure 8.**
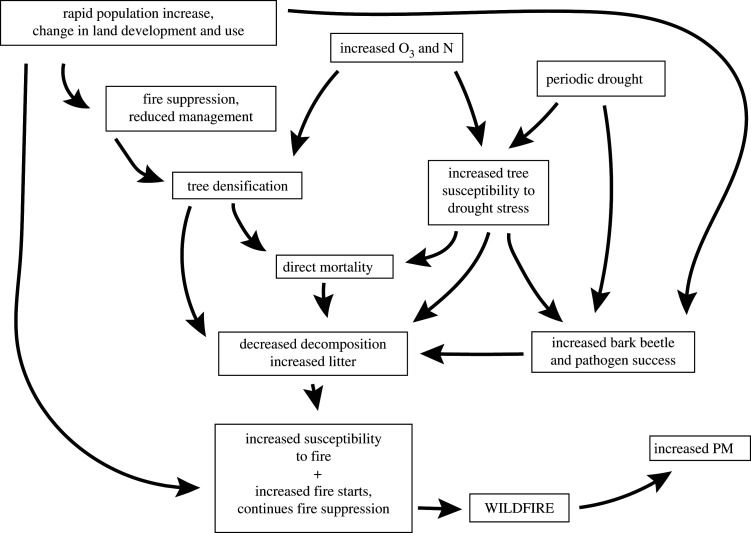
Conceptual diagram showing the interactions, effects and feedbacks of human management and ozone and atmospheric nitrogen deposition on forest processes and susceptibility to wildfire and production of airborne particulate matter. Modified after Grulke *et al*. [140].

Finally, the occurrence of wildfires has increased over recent decades, and with these events a deterioration in air quality, largely due to increases in fine PM [18,21]. Many factors influence the susceptibility of forests to wildfire, including increases in local human population and associated development adjacent to forest lands, fire suppression, and warming and drought. Increasing fire frequency and severity are linked to climate change [138]. In addition to these factors, research has demonstrated a linkage between air quality and forest predisposition to wildfires, which has been manifested in southern California [139]. Increases in atmospheric nitrogen deposition and ozone shift the processing of water, carbon and nitrogen in forest ecosystems, resulting in a cascade of synergetic effects, which make trees more prone to disease, pest invasion, drought and ultimately wildfire (figure 8). These air pollutants increase leaf turnover and litter mass, and decrease the decomposition of litter [141]. As a result, mixed conifer forests of southern California that are impacted by nitrogen and ozone pollution develop deep litter layers. Under high ozone and nitrogen, trees also shift their allocation of biomass away from foliage and roots. Elevated ozone decreases plant control of water loss increasing transpiration, which when coupled with loss of root mass increases the susceptibility of trees to drought stress. Ozone and drought stress and enrichment of nitrogen makes trees particularly vulnerable to attack by bark beetles [142]. The enhanced fuel load from tree decline and litter accumulation driven by nitrogen and ozone pollution coupled with development pressures and climate have resulted in catastrophic fires in southern California in recent years, exacerbating already severe air quality and health impacts. Such feedbacks will continue to influence wildfires, and thereby accelerate air quality and the associated impacts in future.

It is important to recognize that impacts of air pollution and climate on ecosystems are not always negative, indeed the few studies that have evaluated ecosystem responses to air pollution and climate change have often shown both positive and negative effects. For example, the first case study discussed above showed that climate warming coupled with decreases in acid deposition have mitigated air pollution damage to red spruce in eastern North America. Moreover, modelling studies have projected increases in net ecosystem production associated with a combination of increases in air temperature that increase the duration of the growing season and atmospheric CO_2_ and elevated atmospheric nitrogen deposition [45,143] which result in fertilization effects on vegetation production. These beneficial impacts may be offset by loss of soil carbon due to increases in mineralization associated with increases in temperature, sometimes increases in nitrate leaching and nitrous oxide production and changes in soil moisture and watershed hydrology.

An important approach used to inform and guide air quality management of ecosystems is critical loads. A critical load is the amount of atmospheric deposition of acidity, nutrients or contaminants (usually expressed as mass per area on an annual basis) below which there is no known ecological harm under future steady-state conditions based on current scientific knowledge [144]. Similar to a critical load, a target load can be determined for a particular timeframe by which the specified level of protection will be attained. Critical and target loads have been widely used for management of air pollution effects in Europe and North America using empirical relationships and steady-state and dynamic models [145,146]. Given the impacts of climate change on ecosystem structure and function, future critical load and target load assessments should incorporate climate change effects into the determination of critical/target loads.

## 4. The need for linkages among sector effects

We (as a research community) address air pollution effects traditionally by sector. Holistic assessments of actual projected costs and/or feedback effects of air pollution across ecosystems, agriculture and human health have to date not been conducted. A joint protocol for a multi-model, multi-effects project and/or a holistic assessment report could help close this gap. The benefits of air pollution controls documented in the scientific literature are overwhelmingly focused on the mitigation of health outcomes. But recent efforts have started to address the co-benefits of improved air quality for health and agriculture, finding that the combined air quality co-benefits for human health and agriculture will counterbalance the implementation costs of measures required to meet the Paris Agreement pledges [58]. One difficulty limiting more holistic considerations is that ecosystem benefits and damages are difficult to monetize. Nevertheless, there are compelling reasons to link the assessment of air pollution and climate stress across sectors. As we and others have suggested previously, air quality and climate management/policy should be linked or at least considered in a holistic way, and should also be viewed as a multi-media problem. Under a changing climate the pathways of air pollution on human health are shifting from predominately direct atmospheric exposure to growing recognition of the importance of indirect multi-media influences from a variety of interconnected environmental factors. This is probably most prominent for mercury (see the example above). Human exposure to mercury as methylmercury largely occurs through the consumption of fish [147] or rice [148]. The transport, methylation and trophic transfer of mercury will all likely be enhanced under climate change, particularly in wet ecosystems [129,149].

There is a climate penalty for ozone, but this climate penalty will likely be accelerated due to temperature-enhanced increases in biogenic VOC emissions [16]. While exposure to ozone reduces wheat yield, the protein proportion of wheat under such conditions has been shown to increase, a possible trade-off implication for nutrition and human health [150]. As discussed above, the contribution of wildfires to atmospheric particulate matter concentrations is increasingly recognized as a component of the air quality climate penalty exacerbated by local air pollution.

Increases in atmospheric deposition of nutrients could be beneficial for crop production [116]. However, it is increasingly recognized that harmful algal blooms from cyanobacteria and red tides that release liver and neurotoxins are becoming increasingly prevalent with increases in air and water temperature and nutrient inputs to lake and coastal marine ecosystems [151]. Chapra *et al*. [152] modelled the future impacts of harmful algal blooms under RCP 4.5 and RCP 8.5 for the coterminous USA, finding significant increases largely due to temperature changes but also nutrients associated with increases in population and increases in runoff. The number of days per year and areal occurrence of harmful algal blooms are projected with increase from RCP 4.5 to RCP 8.5 scenarios and through this century. The greatest increases in occurrence are projected for the northeastern USA, while the greatest costs due to impaired recreation occur in the southeast. Atmospheric deposition of nutrients (nitrogen, and to a lesser extent phosphorus), in addition to land runoff, fuel harmful algal blooms [153,154]. In addition, emission controls on sulfur to improve air quality and reduce acid rain have led to reduced atmospheric sulfur deposition and increased the need for sulfur fertilizer application [117]. Going forward, models of changes in atmospheric deposition under changing climate should be coupled with ecosystem models to project the simultaneous impacts of these long-term changes.

## 5. Conclusion and recommendations

The largest body of literature that evaluates the impact of future air quality projections on human and natural resources in a systematic way is focused on human health. While published research generally addresses these sectors in isolation, there have been initial studies examining effects of future air quality on both human health and crops (e.g. [58]). For these two sectors, there is significant overlap in models and scenarios used to evaluate future projections. This is, however, not the case for ecosystems. While many studies evaluate scenarios of future air quality effects on ecosystems [47,48], fewer have coupled these with future climate scenarios [155,156]. Given the similar time scale of responses of ecosystems to atmospheric deposition and climate change and the marked responses of ecosystem hydrology, biogeochemistry, and plants and animals to changing climate, it is critical that climate impacts be integrated into assessments of the impact of air pollution on ecosystems.

Of the literature evaluating projected effects of future air quality on human health, studies generally show that the level of health effects projected are strongly dependent on the level of ambition. Substantial health benefits for moderate to low emission RCPs and more stringent policy scenarios (e.g. MFR, attainment of the 2°C target) are projected. Health benefits from reduced PM burdens are projected to outweigh those from reduced ozone globally by more than a factor of 10 (e.g. [50,54]) and global health benefits are driven largely by changes in Asian emissions. Health benefits emerging following reduced Asian emissions manifest strongly at the local/regional level but are also documented to propagate to neighbouring countries and hemispherically [69]. Recent work has also highlighted substantial health benefits emerging from cap and trade policies and/or technological innovations/transitions in the transportation and energy sector (e.g. [62,63,157]). For those studies that consider factors such as population and demographics, they indicate that population growth and an ageing population will exacerbate health effects with larger populations being exposed to unhealthy air pollution at the regional level. Whether global or regional studies, almost all studies indicated that the cost of the policy implementation would be largely compensated for by or in most cases far exceeded by the monetary health benefits (on the order of millions to trillions of USD depending on the geographical scope of the study and level of ambition in the scenario). Consistent with global health benefits being driven by changes in Asian emissions, the associated monetary benefits are also larger in Asia (e.g. [55,58,64,107]).

Globally, ozone damage to crops is expected to result in approximately 10% or less additional production loss by 2030/2050 under high emissions (RCP8.5/6.0) scenarios. Reduced emissions (RCP4.5, 2°C target) scenarios can lead to small global crop production gains of a few per cent. Changes to future production are expected to be larger in several regions, including India and China, but these are also more widely varied due to uncertain emissions trajectories. The impacts of PM on crop production are rarely studied and highly uncertain, but the future reduction of PM under reduced emissions scenarios could lead to global production loss of several per cent. The potential decrease in crop production owing to a decrease in PM is, however, much less significant than the likely improvement in human health associated with these reductions. As such, projected effects should be considered holistically and not in silos. Recent work points toward the use of large observational datasets to derive spatially varying relationships, rather than static CR functions, to better represent processes such as regional adaptation to pollution. The few studies that quantified the economic costs of crop losses globally under A2, B1 and CLE scenarios, comparing 2000 to projections for 2030, were relatively consistent, indicating costs ranging from 12–35 billion USD annually [105,106]. These costs are large enough to offset a significant portion of expected GDP growth rates, especially for those countries that are largely agriculturally based.

Climate is fundamental to the structure and function of ecosystems. Air pollution involves a host of contaminants (e.g. ozone, sulfur, nitrogen, mercury) and can acidify, eutrophy and/or increase toxic conditions of ecosystems through direct or indirect effects. The effects of climate change on ecosystem structure and function can either mitigate or exacerbate air pollution effects. The response of ecosystems to air pollution and climate change is long-term, complex and varies widely across biomes and over space and time. Although models have been effectively used to project the long-term response to changes in emissions and atmospheric deposition or air quality, few studies have investigated the future response of ecosystems to air quality changes in conjunction with climate change, such as the RCP scenarios. Given the multi-decadal or century long time frame of air pollution and climate impacts, future assessments of air pollution effects on ecosystem structure and function need to recognize and accommodate the interactive effects of these disturbance regimes.

The complex, interlinked nature of air pollution effects on diverse endpoints argues for a more holistic assessment of these effects, not only to improve our understanding of how (potential) policy decisions affect these different endpoints, but also to better capitalize on synergies. One possibility for a holistic assessment across sectors would be a joint protocol for a multi-model, multi-effects project, established as an explicit interdisciplinary effort. Since expertize across multiple sectors is rare, an interdisciplinary collaboration would allow for an appropriate application of methods and associated quantification of uncertainty, as well as an effort to standardize expression of the quantified effects. The final step to affect quantification could be the use of a common unit of output, such as economic cost, to facilitate comparison across sectors and allow for a comprehensive cost-benefit analysis, recognizing that monetizing certain types of benefits may be difficult and associated with substantial uncertainty. Given that studies of individual sectors show that the benefits of mitigation policies generally outweigh the costs, a comprehensive analysis would likely show even greater benefits. An envisioned outcome would be to foster the implementation of more ambitious mitigation policies that more clearly characterize and quantify co-benefits associated with reducing adverse effects of air pollution and climate change across human health, agriculture and ecosystems in the future.

## Data accessibility

This article has no additional data.
